# Structural, Functional and Neurochemical Cortical Brain Changes Associated with Chronic Low Back Pain

**DOI:** 10.3390/tomography8050180

**Published:** 2022-08-25

**Authors:** Yara Medrano-Escalada, Gustavo Plaza-Manzano, César Fernández-de-las-Peñas, Juan Antonio Valera-Calero

**Affiliations:** 1 Clínica FisioAvanza Delicias, 28045 Madrid, Spain; 2 Department of Radiology, Rehabilitation and Physiotherapy, Universidad Complutense de Madrid, 28040 Madrid, Spain; 3 Grupo InPhysio, Instituto de Investigación Sanitaria del Hospital Clínico San Carlos (IdISSC), 28040 Madrid, Spain; 4 Department of Physical Therapy, Occupational Therapy, Rehabilitation and Physical Medicine, Universidad Rey Juan Carlos, 28922 Alcorcón, Spain; 5 Clínica e Investigación en Fisioterapia, Terapia Manual, Punción Seca y Ejercicio Terapéutico, Universidad Rey Juan Carlos, 28922 Alcorcón, Spain; 6 VALTRADOFI Research Group, Department of Physiotherapy, Faculty of Health, Universidad Camilo José Cela, Villanueva de la Cañada, 28692 Madrid, Spain; 7 Department of Physiotherapy, Faculty of Health, Universidad Camilo José Cela, Villanueva de la Cañada, 28692 Madrid, Spain

**Keywords:** low back pain, cortical brain changes, neuroscience, chronic pain, neuroimaging

## Abstract

Chronic low back pain (CLBP) is one of the most prevalent musculoskeletal disorders, being one of the leading contributors to disability worldwide and involving an important economic and social burden. Up to 90% of CLBP is non-specific (not associated with specific injuries), with a chronicity expectation estimated at 10%. Currently, motivational and emotional central circuits are being investigated due to their role in CLBP persistency and chronification. Therefore, this narrative review aimed to summarize the evidence regarding the cortical brain changes described for proposing novel multidisciplinary approaches. Novel advances in neuroimaging techniques demonstrated structural (e.g., decrease in the grey matter located at the dorsolateral prefrontal cortex), functional (e.g., connectivity impairments in those areas involved in pain processing), and neurochemical changes (e.g., decrease in cerebral metabolites). In addition, significant changes were found in the primary somatosensory and motor cortex, contributing to the alteration of low back muscles activation and function.

## 1. Background

Low back pain (LBP) is defined as the musculoskeletal syndrome characterized by a set of symptoms (being the presence of pain focused on the final segment of the spine the main one) in the lumbar area, located between the lower ribs and the sacral region [1]. Clinically, it can be presented as acute pain episodes if <4 weeks of duration, subacute if 4–12 weeks of duration or chronic if >3 months of duration [2]. Chronic low back pain (CLBP) has a more complex nature compared with acute episodes since cognitive, emotional, behavioral and social factors directly affect the CLBP experience [3]. In fact, evidence is consistent regarding the role of anxiety, depression, catastrophism, kinesiophobia and somatization as risk factors of LBP chronification [4].

LBP is the main cause of disability worldwide [5], with 80% of the population suffering from it at some point in their lives. Most of the people can expect to be recovered in 4–6 weeks (50–80%), but more than half have the possibility of relapse in the following 12 months. In addition, up to 10–15% the pain can become chronic [5]. Thus, a high percentage of these patients do not respond to treatment, causing limitation in functional capacity, suffering emotional disturbances derived from the constant state of pain.

Resources dedicated to LBP management are not negligible. For instance, in Spain, +1.2 M patients seek medical attention in a 6-month period, involving an economic burden estimated at EUR 16,000 M a year (comparable with other conditions, including cancer or cardiovascular diseases) and social impact (up to 40% of working absenteeism is caused by LBP) [6].

Approximately in 80–90% of the patients suffering LBP, pain is not associated with a specific injury, such as fracture, trauma, systemic diseases or root compression [7]. This clinical picture is defined as non-specific low back pain. The 10–20% left is associated with red flags. Red flags are characteristics of the patient’s medical history and physical examination which are considered to be associated with a higher risk of serious pathology associated with patients’ complaints, needing referrals for medical attention [8].

It should be noted that the probability of X-ray to identify the cause of back pain is less than 1%. In a previous study conducted with LBP patients [9], no abnormalities were found in 65% of the radiographs. The prevalence of degenerative changes was high (>62%) in patients aged 55 years or older, and the prevalence of possible tumor was low (<1%). This leads us to suspect that the use of radiography in LBP results in a substantial increase in cost and a risk due to the increase in radiation to patients in relation to the benefits [10].

Therefore, since LBP is considered the musculoskeletal condition with the highest prevalence (14.8% acute LBP and 7.7% chronic LBP), which causes a significant decrease in the patient’s and family’s quality of life [11], the aim of this narrative review is to provide an overview of neurochemical, structural and functional changes in the brain cortex associated with LBP.

## 2. Imaging Methods for Assessing Cortical Brain Changes

Neuroplasticity (defined as “the ability of the nervous system to change its activity in response to intrinsic or extrinsic stimuli by reorganizing its structure, functions, or connections”) has positive characteristics (known as adaptive), which may also become negative (maladaptive) [12]. Because of these maladaptive neuroplastic changes in chronic LBP, several methods for assessing brain properties have been used and developed.

- Transcranial magnetic stimulation (TMS): Represents a painless and non-invasive technique for investigating the integrity and function of the corticospinal pathway and the primary motor cortex (M1). A magnetic body on M1 can depolarize the corticospinal cells by which, at a sufficient intensity, the stimulus produces a muscular response which is called motor evoked potential (MEP), which is recorded by electromyography electrodes. The PEM latency and amplitude are considered primary outcomes for corticospinal function testing [13].
- Voxel-based morphometry: This brain morphological imaging method uses 3D magnetic resonance and allows the measurement of gray matter (GM) volume and morphological and structural brain changes detection. The intensity of these changes is shown to be negatively associated with the duration of pain, and the tendency in chronic LBP is toward a decrease in gray matter [14].
- Functional magnetic resonance imaging (fMRI): It is a non-invasive medical examination. It is based on a powerful magnetic field for observing small changes which occur in brain (particularly, changes in the cerebral blood flow). The BOLD signal is an indirect marker of neural activity. It is used to examine the functional anatomy and perform a brain mapping (determining which part of the brain is controlling essential functions). The most evaluated areas using fMRI in patients with CLBP are the primary and secondary somatosensory cortex (S1 and S2), the anterior cingulate cortex (ACC), the prefrontal cortices and the thalamus [15].
- Arterial spin labeling: This is the fMRI technique based on measuring cerebral perfusion (uses water in arterial blood as a free diffusion marker) non-invasively. It allows the absolute quantification of regional cerebral blood flow, whose image may be more accurate than BOLD and seems to be one of the most appropriate tools for studying chronic pain experience features [16].
- Magnetic resonance spectroscopy: It is a non-invasive brain imaging method used for exploring metabolic concentrations in certain regions of the brain. It detects radiofrequency signals generated by the magnetic nuclear spins of magnetically active nuclei such as protons, phosphorus, carbon, and fluorine, which are excited by external magnetic fields. Some changes in the concentration of metabolites (N-acetyl-aspartate (NAA), creatine or glutamate) that have been found in several patients with chronic pain are represented, suggesting biochemical brain alterations in chronic pain [17].
- FreeSurfer: A set of tools for neuroimaging analysis through algorithms to quantify the functional and structural properties of the brain. It automatically creates models of the macroscopically visible structures in the human brain, given a reasonable T1-weighted input image [18].
- Diffusion-weighted (DWI) and diffusion tensor imaging (DTI): This magnetic resonance base allows the exploration of the micro and macro architecture of the brain, allowing the understanding of tissue injury in vivo, the development of white matter tracts and functional connectivity within the brain [19]. DWI can produce multiple informative metrics at each voxel, including fractional anisotropy (the degree of diffusion restriction to reflect white matter integrity), apparent diffusion coefficient (a measure of the magnitude of diffusion of water within a tissue, and can be used in monitoring brain infarctions), axial diffusivity and radial diffusivity (diffusion rates along the main and transverse diffusion directions respectively). Among the different signal models, DTI is one of the most popular [20]. DTI provides information about the covariance structure of the molecule diffusion displacement distribution, which is related to the directionality of the water diffusion process. Therefore, this method is characterized by high sensitivity to small macro and microstructural changes in white matter tissue. The trace images provide information about the magnitude of diffusion, and the shape of the diffusion tensor may change independently from the overall size or magnitude of the diffusion tensor [19].

## 3. Cortical Changes in Chronic Low Back Pain

Multiple mechanisms contributing to the transition from acute to chronic pain have been proposed, involving both the peripheral nervous system and the central nervous system (CNS). Although the state of the brain in chronic pain has a role yet to be clarified, it is widely accepted that we cannot think of chronic pain as an input of nociceptive stimuli into a brain that is functioning correctly. Therefore, neuroplastic remodeling can lead to the maintenance of pain over time, even in the absence of nociceptive input [21].

Neuroimaging studies have revealed numerous structural and functional changes in the brain of those with chronic musculoskeletal pain. These changes can be broadly classified as neurochemical, structural, or functional.

### 3.1. Neurochemical Changes

The detection of neurochemical brain alterations by magnetic resonance spectroscopy in the presence of persistent pain allows the scientific community to detect changes associated with specific pain situations for better understanding of the underlying mechanisms, developing potential therapies and aids in the diagnosis of painful disorders. Significant changes (either increased or decreased depending on the marker) have been observed at the thalamus, dorsolateral prefrontal cand orbitofrontal cortices, being a differentiator factor between healthy subjects and patients with chronic LBP [22]. In fact, the magnitude of the changes found were directly proportional to pain intensity and duration. Although it is not possible to prove whether neurochemical changes induce chronic LBP, evidence seems consistent regarding the neurochemical profile variations induced by this condition [23].

A systematic review conducted by Zhao et al. [24] summarized the biochemical changes in brain regions of interest and the hypothesized possible mechanisms explaining those findings. Nine studies meeting the inclusion criteria were included, recruiting a total of 135 subjects with chronic LBP and 137 healthy controls. Brain metabolites were studied through magnetic resonance spectroscopy (i.e., N-acetyl-aspartate (NAA), choline, creatine, glutamate, glutamine, gamma-aminobutyric acid (GABA) and glucose), finding significant differences between cases and controls in specific brain regions, such as the thalamus, insula, S1, dorsolateral prefrontal cortex (DLPFC), ACC and primary motor cortex (M1).

NAA is present in very high concentrations in brain neurons and has been recognized as a brain marker. This metabolite is reduced in various brain regions in patients with chronic LBP as in neurodegenerative diseases, demonstrating the correlation between neuronal loss and degeneration [25]. As suggested by Apkarian et al. [22], a cerebral atrophy in the thalamus, S1 and DLPFC could be related to the NAA decrease. In addition, myo-inositol (which is present in glial cells) is significantly reduced in the ACC and thalamus in patients with chronic LBP [26].

Regarding glutamate, which is the most abundant excitatory neurotransmitter in the brain, one study showed a decrease in the ACC in patients with chronic LBP [26]. This finding is controversial with the results reported by other studies [27], which through proton magnetic resonance spectroscopy, detected an increase in glutamate (which in high concentrations has an excitotoxic effect that can lead to cell death of glutamatergic neurons), which play a key role in pain processing [28].

Finally, GABA is the main inhibitory neurotransmitter, and its receptors are found in the thalamus, spinal cord and cortex [29]. A decrease in GABA levels by selectively activating GABA(B)-receptor-bearing rostral agranaular insular cortex neurons at the insula increases pain through projections to the amygdala, while preventing its degradation by using an enzyme inhibitor or gene transfer mediated by a viral vector relieves it by enhancing the descending inhibition of spinal nociceptive neurons, suggesting its key role in the pathophysiology of some chronic pain conditions [28]. In contrast, the study by Zhao et al. [24] found no significant changes in the GABA neurotransmitter in patients with chronic LBP compared to healthy subjects, which may be due to extremely low concentrations and the overlapping of signals from other brain metabolites.

### 3.2. Structural Changes

Current evidence suggests a gray matter reduction in the DLPFC, right anterior thalamus, S1, S2, posterior parietal cortex, left temporoparietal junction, superior frontal gyrus, right frontal pole and middle cingulate cortex in people with chronic LBP since results demonstrated a strong correlation between the density changes magnitude with pain intensity [22,30,31,32,33,34,35,36,37].

LBP cannot be understood just as an altered functional state. Chronic LBP is also a consequence of neuroplastic changes and structural brain reorganization, altering the processing of nociceptive sensory information. Apkarian et al. [21] examined brain morphometry (through voxel-based morphometry) under chronic pain conditions and concluded that subjects with chronic LBP suffer a greater neocortical gray matter atrophy than the normal atrophy attributable to age in healthy populations.

Regarding the volume of cortical gray matter, the difference found between patients with chronic LBP compared with healthy subjects was 5.4% smaller (which is equivalent to 30 cm^3^). It should be noted that a decrease of 0.5% per year (2.8 cm^3^) was attributable to aging in both groups, and controlling for affect, age and medications could reduce or eliminate these GM volume alterations found in CLBP [35]. In addition, a local decrease in gray matter density was observed bilaterally at the DLPFC in subjects with chronic LBP compared with controls. However, there were no differences within the three regions of the DLPFC assessed in the study. This decrease in density was significantly correlated with pain-related measures (pain intensity, pain duration, sensory and negative–affective dimensions of chronic LBP), age and gender, but not with anxiety or depression [22]. Similarly, although Ivo et al. [30] found significant decreases in GM density at the DLPFC, thalamus and middle cingulate cortex, no correlations between structural data with anxiety or depression were found.

On the other hand, the thalamus revealed decreased gray matter in the right anterior area (which is responsible for mediating nociceptive inputs to the cortex, hypothesizing that thalamocortical processes may play an important role in the pathophysiology of chronic pain) [30].

The regional pattern of atrophy appears to be specific to chronic pain, as it affects regions involved in pain perception and may explain the transition from acute to chronic stage [21]. Chehadi et al. [34] using Voxel-based morphometry revealed the negative correlation between though suppression (which is a common type of cognitive control of pain) and GM volume in the left superior and left middle temporal gyri (both associated with pain intensity).

Apkarian et al. [22] also analyzed chronic LBP patients with neuropathic pain components (significant radiculopathy in the leg) and patients without neuropathic pain. The DLPFC gray matter density decrease was significantly greater in subjects with neuropathic pain (27%) compared with those subjects without the neuropathic component (14%). This may be related to the more negative and debilitating effect of neuropathic pain in this subgroup of patients with chronic LBP.

Kong et al. [31] performed a neuroimaging study in healthy subjects and subjects with chronic LBP seeking for morphometric and volumetric differences through T1-FreeSurfer sequence exploration. The study focused on thickness differences located at S1 (specifically, the upper third, which, according to Penfield’s sensory homunculus, captures the cortical representation of the lower back). The comparison between patients with chronic LBP and the control group showed that the cortical thickness measurement of the postcentral gyrus (S1 area) was bilaterally greater in those subjects with chronic LBP. Thus, volume increase was observed in the upper third of S1 and not in the middle or lower third, which highlights the specificity of the changes in the representation of the lower back. In addition, these changes can be related to hypersensitivity of the CNS in patients with chronic LBP (characterized by lower pain threshold and lower pain tolerance values) compared with healthy subjects.

Finally, Ung et al. [32] carried out one study aiming to differentiate patients with chronic LBP based on structural changes in the brain and to investigate pathological changes in certain regions of the cortex. They found a pattern of regional gray matter density that distinguished, with 76% accuracy, patients with chronic LBP from healthy controls. The most notable changes were an increase in gray matter in the left S1 and S2 cortices, left M1, and premotor cortex. Teutsch et al. [33] found a gray matter increase in regions processing pain modulation (especially in S1), transiently in healthy subjects subjected to a repetitive noxious stimulus. This adaptation may be related to the compromise of a normal antinociceptive system, but in patients with LBP, the increase (especially in S1), may be the result of a defective antinociceptive system due to a noxious stimulus sustained over time, indicating that the pain no longer depends on peripheral afferent input but on central processing.

### 3.3. Functional Changes

Assessing functional connectivity in the brain refers to the temporal dependence of neuronal activity between anatomically separated regions, it being essential to carry out cognitive processes by integrating information. The brain’s default mode network (BDMN) is the first network to show functional connectivity in a non-task resting state and is believed to play a fundamental role in the synchronization of all brain regions [36,37].

Kregel et al. [38] described altered functional connectivity in patients with CLBP during rest and increased activity in pain related areas following painful stimulation in several brain regions, especially those associated with pain dimensions (known as the “pain matrix” formed by the prefrontal cortex, cingulate cortex, insula, thalamus, S1 and S2 cortices). Wasan et al. [16] experimentally induced pain in chronic LBP patients and healthy subjects, finding regional blood flow changes in certain brain areas, especially those referenced in the pain matrix. Pain exacerbation was performed in 16 patients with chronic LBP and 16 healthy subjects, using two methods: heat and clinical maneuvers. Only the clinical maneuvers, leg elevation or pelvic tilt, accounted for a significant increase in pain from the clinical point of view, being greater than 30% of the initial pain. This increase in endogenous pain was associated with changes in cerebral blood flow in certain cortical areas such as the medial and DLPFC, S1 and S2 cortices, right insula, and superior parietal lobes in patients with chronic LBP. Although the latter are not part of the pain matrix, they have a high functional connectivity with the rest of the areas in pain states.

On the other hand, Matsuo et al. [39] aimed to examine the dysfunction of descending inhibitory paths mechanisms in subjects with chronic LBP through fMRI in a 3-Tesla MRI scanner. They recruited 11 patients and 13 healthy subjects who discontinued pharmacological treatment 24 h before the study participated. A mechanical stimulus of 500 kPa was applied for 30 s in 3 blocks, with 30 s rest, reaching a painful level for the participants who were asked to remember. The most notable findings were that chronic LBP patients lacked activation in the ACC and DLPFC after pain stimuli, while clear activation of these regions appeared in healthy controls. ACC and DLPFC mediate the affective and cognitive components of pain perception and are also believed to be the cortical origin of the descending inhibitory system.

Baliki et al. [40] studied brain activity associated with spontaneous pain and acute thermal pain in patients with chronic LBP (which is one of the main complaints of these patients). They analyzed 13 patients with chronic LBP suffering spontaneous pain through fMRI differentiating two periods; one in which the spontaneous pain is maintained at a high intensity and another in which the spontaneous pain increases transiently, and they were able to determine which different regions of the brain are responsive in each of the phases. In high but sustained spontaneous pain, they observed activity at the medial prefrontal cortex (MPFC) and ACC with projections to areas of the brain involved in emotions, cognition, and motivation (i.e., posterior thalamus and the amygdala). In contrast, when spontaneous pain is exacerbated, the brain activation pattern observed corresponds to that observed in acute pain, encompassing regions involved in sensory and affective dimensions of pain (i.e., right insula, S1, S2 and cerebellum).

In addition, they found a significant relationship between increased brain activity in MPFC in situations of high intensity pain in phase 1 (high but sustained pain), and in the right insula with a longer duration of pain in phase 2 (transient increase in spontaneous pain). These findings explain whether the activity in the insula reflects the chronicity of LBP and the activity in the MPFC reflects its intensity. The response to thermal stimuli activated, in both the controls and patients, the insula bilaterally, S2, cingulate cortex and DLPFC, which made it clear that spontaneous pain does not activate the same brain regions as an external stimulus. It was observed a dissociation between the emotional and sensory regions in terms of encoding pain intensity in acute pain, compared to sustained spontaneous pain. Whereas the sensory region (insula) encodes the perceived magnitude of acute pain, the emotional region (MPFC) encodes the magnitude of spontaneous pain in chronic LBP.

According to Apkarian et al. [22] and Baliki et al. [40], a decrease in gray matter can cause its dysfunction and consequently an increase in the activity of the MPFC, which reflects a negative emotional state caused by chronic pain. This provides a link between brain atrophy and continued suffering from chronic low back pain. A subsequent study by Hashmi et al. [41] hypothesized that the representation of LBP over time moves away from sensory areas and gradually becomes involved in emotional and limbic regions. Patients with subacute low back pain were observed and divided into one of two groups over a year: (1) those who had recovered and (2) those who had progressed to chronic pain. They were also compared with patients with chronic LBP of longer duration. The results obtained in fMRI determined that the perception of low back pain in patients with chronic LBP over 10 years did not activate the same brain circuits as in those with LNP of 2 months of duration. The latter activated circuits more related to acute pain and reward, (i.e., insula, thalamus, S1, and ACC), whereas in subjects with chronic LBP, emotional circuits were activated (i.e., amygdala, MPFC, orbitofrontal cortex and hippocampus).

Changes observed in subjects with persistent pain at 1 year compared to baseline activity were a significant increase in functional connectivity between the MPFC and the nucleus accumbens. This argues that brain activity in the transition from acute to chronic pain shifts away from sensory circuits to increase activity in emotion circuits (mesolimbic circuits). The first year may be critical for this pain chronification since it was observed that patients with persistent subacute pain (1 year) had similar activity to patients with chronic LBP lasting more than 10 years, which proposed a time window for the stabilization of chronic pain from 6 to 12 months, which suggests that the best form of intervention to prevent the chronification of low back pain is before the first year [41].

Li et al. [42] assessed the association between gray matter volume changes and functional changes in the cerebral cortex in patients with chronic LBP. They recruited 16 chronic LBP patients and 16 healthy subjects, inducing a mechanical painful stimulus at the left lower back, and analyzed the brain structure and functional connectivity through voxel-based morphometry and fMRI, respectively. The results obtained were consistent with previous findings, observing a decrease in gray matter in the DLPFC, thalamus, ACC and MPFC, areas involved in nociceptive and affective/cognitive processing, and an increase in gray matter in areas related to sensory information (S1 and M1). They also found disrupted default neural network functional connectivity and increased connectivity after painful stimuli in S1, S2, cerebellum, and insula. The increase in gray matter in S1 and M1 corresponded to the somatotopic representation of the trunk, which shows causality with the pain located in the lower back. On the other hand, connectivity between bilateral sensorimotor areas and the superior parietal lobe (which is the area of sensory integration) was increased in subjects with chronic LBP compared with healthy subjects, and was positively correlated with pain intensity. This increase in the activity of S1 and M1 may lead to an increase in gray matter in the region of the trunk of S1 and M1, which can be considered a sign of chronic low back pain. These current results show an insight into the influence of the interaction between the structural reorganization of the brain and the functional changes that may be the basis of the chronification of low back pain.

Regarding the motor cortex and muscular activation changes found, a large number of studies reported that people with chronic LBP plan movement differently, and especially have post-activation of trunk muscle contraction during rapid upper extremity movement (i.e., a delayed early postural adjustment). Due to the participation of cortical structures, such as M1, the supplementary motor area, cerebellum or basal ganglia, in the execution and planning of the anticipated postural adjustment, and since the muscles of the spine are controlled by corticospinal pathways, it is logical to think of the relationship that exists between motor alteration and plastic changes in M1 and other cortical motor areas [13].

Tsao et al. [43] observed the reorganization and excitability of the motor cortex present in the changes of the postural activation of the transversus abdominis (TrA) muscle in 11 patients with chronic LBP and 11 healthy subjects. They asked study subjects to perform rapid arm movements during a trunk-altering task to assess postural activation of TrA. The results associated the reorganization of motor cortex networks with delays in TrA activation, as well as a change in its cortical representation, as we have already mentioned. The magnitude of the representation change in the motor cortex was related to the magnitude of the delay in postural activation of TrA. Activation of the deep abdominal and trunk muscles (especially TrA) occurs prior to deltoid activation in voluntary limb movements, and that prior contraction is controlled by the CNS. The problem lies in the fact that patients with chronic LBP have a delay in the activation of TrA, and those other lumbar muscles, such as the multifidus, are also affected. It is suggested that this reorganization in the motor cortical map of TrA and the rest of the muscles can distort the coordination between the trunk muscles in subjects with chronic LBP, increasing their pain and disability.

A review of the literature carried out by Massé-Alaire et al. [40] highlighted the link between pain-related brain reorganization and altered TrA control. They observed that the ACC, thalamus and MPFC in patients with chronic LBP present biochemical changes and hyperexcitability. The ACC participates in motor planning and is directly connected to M1 and the supplementary motor area, both of which are involved in automatic postural adjustment, which is impaired in subjects with chronic LD. They also observed a reorganization of the sensory maps with medial displacement of S1. All of this neuroplasticity may be related to proprioception and tactile acuity deficiency underlying observed motor changes. Chronic LBP causes a decrease in intracortical inhibition of the pain area in M1, which means an alteration in neuronal homeostasis with changes in the cortical maps between neighboring neuronal networks. Specifically, in the LBP, corticospinal excitability corresponding to the painful region, the low back, decreases with increasing pain and disability, and this can be explained by a change in the motor maps of M1, the posterolateral deviation of the representation of TrA, related in amplitude to the delay of TrA activation.

As Massé-Alaire described in another study [13], changes in the representation of S1 may have a fundamental role in the relationship between pain and impaired motor control of movement, given its substantial role in both sensory encoding and aspects sensory-discriminative of pain. S1 and M1 have reciprocal connectivity, which implies that a displacement in the representation of the trunk in S1 can affect the connectivity with M1 and, consequently, a lower performance in motor control of the spine in patients with chronic LD. Additionally, altered neural processing, supplementary motor area connectivity, and cerebellar connectivity and density changes are involved in postural control and anticipatory postural adjustment through transcortical and cerebellar-cortical connections with M1 areas. They also stated that the plasticity of M1 is greater in patients with severe chronic LBP than in those with moderate or mild chronic LBP [13].

Čeko et al. [44] examined whether CLBP affects connectivity of brain networks supporting cognitive functioning and changes after treatment. They found bilateral insula as the region of aberrant cognitive resting-state connectivity comparing patients and controls with fMRI and structural changes in white matter with DTI. In addition, previous studies [45,46] also observed reduced fractional anisotropy values in the corpus callosum, bilateral anterior thalamic radiation, right posterior thalamic radiation, right superior longitudinal fasciculus and left anterior corona radiate associated with the abnormal functional connectivity, damaged white matter tracts, altered resting-state functional connectivity of bilateral thalamo-motor/somatosensory pathways and impaired empathic abilities in patients with CLBP. However, brain function seems to restore partially after treatment [45].

Therefore, these brain plasticity findings contribute to a better understanding of the pathophysiological mechanisms in patients with CLBP and clarify whether the induction of positive plasticity changes promoting function and reducing pain should be considered during CLBP rehabilitation. Clinicians should consider therapeutic strategies for normalizing S1 and M1 maps. Sensorimotor integration training will favor the activation of sensory recreations and the required motor planning to reinforce synaptic efficiency and cause cortical changes, such as synaptogenesis and the multiplication of dendritic connections [13,44,45,46,47].

Finally, there are some limitations to be acknowledged. Firstly, this is a narrative review and no systematic procedures were followed. Therefore, since relevant studies may not appear in this study, there is a possibility of biased conclusions in this study. In addition, it is important to emphasize the need to pay attention to the removal of the influence of systematic errors in the measurements, which can be large in the case of diffusion-weighted measurements, where the size of systematic errors and their impact on DTI parameters and the possibility of eliminating their impact can be assessed. For instance, although DTI scalar maps emerged as a measure sensitive to tissue structure, they fail to characterize the highly complex diffusion topology, as in the presence of white matter crossing fibers, where the diffusion displacement distribution is multimodal. Thus, DTI suffers from limited biological specificity concerning several microstructural tissue properties within a given voxel (i.e., myelination, axonal packing, axonal orientation dispersion) and is also influenced by multiple non-biological factors, such as the scanner parameters, data quality or head motion [48].

## 4. Conclusions

Chronic LBP should not be considered as a single musculoskeletal pathology. Advances in neuroscience demonstrated that it encompasses CNS, spinal, and muscular neuroplastic changes. For better pain understanding (especially from the patients’ point of view), it is necessary to understand that cortical changes lead a perpetuation of pain, despite having recovered from the initial injury. Current evidence suggests that the passage from acute–subacute to chronic pain is propitiated by the mesolimbic pathways (emotional and motivational) of the brain. Therefore, a clinical correlation between the radiological findings of the lumbar spine with referred pain cannot be expected in chronic LBP.

The cortical brain changes reported in the literature include structural changes (e.g., decrease in gray matter at the DLPFC), functional changes (e.g., modification of the functional connectivity at regions forming the “pain matrix”) and neurochemical changes (e.g., lack or overplus of neurotransmitter in specific areas of the brain). There is also a clear affectation of the M1 area activity, resulting in the function and muscular coordination alteration of the trunk and the abdominal muscles. Therefore, treatment programs should be focused on reversing these changes in order to achieve proper function of the abdominal muscles and the trunk.

## Data Availability

Not applicable.
